# Adolescent behavioral and neural reward sensitivity: a test of the differential susceptibility theory

**DOI:** 10.1038/tp.2016.37

**Published:** 2016-04-05

**Authors:** J S Richards, A Arias Vásquez, D von Rhein, D van der Meer, B Franke, P J Hoekstra, D J Heslenfeld, J Oosterlaan, S V Faraone, J K Buitelaar, C A Hartman

**Affiliations:** 1 Department of Cognitive Neuroscience, Donders Institute for Brain, Cognition and Behaviour, Radboud University Medical Center, Nijmegen, The Netherlands; 2 Karakter Child and Adolescent Psychiatry University Centre, Nijmegen, The Netherlands; 3 Department of Human Genetics, Donders Institute for Brain, Cognition and Behaviour, Radboud University Medical Center, Nijmegen, The Netherlands; 4 Department of Psychiatry, Donders Institute for Brain, Cognition and Behaviour, Radboud University Medical Center, Nijmegen, The Netherlands; 5 Department of Psychiatry, University Medical Center Groningen, University of Groningen, Groningen, The Netherlands; 6 Department of Clinical Neuropsychology, VU University Amsterdam, Amsterdam, The Netherlands; 7 Departments of Psychiatry and of Neuroscience and Physiology, SUNY Upstate Medical University, Syracuse, NY, USA

## Abstract

Little is known about the causes of individual differences in reward sensitivity. We investigated gene–environment interactions (GxE) on behavioral and neural measures of reward sensitivity, in light of the differential susceptibility theory. This theory states that individuals carrying plasticity gene variants will be more disadvantaged in negative, but more advantaged in positive environments. Reward responses were assessed during a monetary incentive delay task in 178 participants with and 265 without attention-deficit/hyperactivity disorder (ADHD), from *N*=261 families. We examined interactions between variants in candidate plasticity genes (*DAT1*, *5-HTT* and *DRD4*) and social environments (maternal expressed emotion and peer affiliation). *HTTLPR* short allele carriers showed the least reward speeding when exposed to high positive peer affiliation, but the most when faced with low positive peer affiliation or low maternal warmth. *DAT1* 10-repeat homozygotes displayed similar GxE patterns toward maternal warmth on general task performance. At the neural level, *DRD4* 7-repeat carriers showed the least striatal activation during reward anticipation when exposed to high maternal warmth, but the most when exposed to low warmth. Findings were independent of ADHD severity. Our results partially confirm the differential susceptibility theory and indicate the importance of positive social environments in reward sensitivity and general task performance for persons with specific genotypes.

## Introduction

Reward sensitivity is an evolutionary important construct; rewards bring about positive feelings, and thereby reinforce the behavior associated with them, enabling learning.^1^ However, under certain circumstances, high reward sensitivity can lead to maladaptive behavior such as increased risk taking in daily life (for example, reckless driving and unprotected sex), substance use disorder and behavioral addictions such as gambling. During adolescence, reward sensitivity is heightened and this may have a key role in the emergence of maladaptive behavior, especially in high-risk groups such as adolescents with attention-deficit/hyperactivity disorder (ADHD).^2^

In the literature reward sensitivity is used as a broad construct referring to the degree in which a person reacts to the mere presence or manipulation of rewards. Indeed, different methods have been used to capture reward-related behavior. For example, by measuring the preference of smaller-sooner rewards over delayed-larger rewards in studies of temporal/delay-discounting,^3^ studying the willingness to perform risky actions in order to obtain rewards,^4^ or by comparing reaction times on rewarded and non-rewarded trials.^5^ Studies focusing on the neural correlates of reward sensitivity/processing have identified various brain regions, in particular the orbitofrontal cortex and striatum, which are activated when receiving or anticipating rewards.^6, 7, 8, 9, 10^ Current evidence suggests that a heightened-responsive neural reward system predisposes to greater reward seeking, whereby increased dopaminergic release in response to rewarding events strengthens reward-related behavior through dopamine-based learning processes.^11, 12, 13^

Reward sensitivity is subject to genetic influences. Studies of delay-discounting have found heritability estimates of 30–51%.^14, 15^ In addition, effects of genes linked to neurotransmitters involved in reward sensitivity, including dopamine, have been reported on several reward-related measures.^16, 17, 18^ Social environmental experiences such as interactions with parents and peers have been associated with behavioral and neural sensitivity to rewards as well. For instance, compared with adolescents with authoritarian parents who make decisions for them, adolescents involved in mutual decision-making with their parents improved on affective decision-making during the Iowa Gambling Task 1 year later.^19^ Similarly, functional magnetic resonance imaging (MRI) studies have revealed associations between low parental warmth and increased responses of prefrontal cortex and striatum to reward anticipation.^20, 21^ In contrast, maternal interpersonal affiliation has been related to increased striatal responses to reward receipt,^22^ and decreased responding of the prefrontal cortex and globus pallidus during reward anticipation has been associated with peer victimization^20^ and childhood adversity.^23^ Although these studies are heterogeneous with regard to how reward sensitivity was operationalized and the environmental variables that were studied, and findings are not consistent in terms of anatomical location and direction of neural activation, they indicate the importance of the social environment in determining the sensitivity to rewards. This is in line with the idea that the social environment has a key role in the development of reward learning. Across development, children's behavior is initially shaped by external rewards such as positive social interactions with parents at first and later with peers as well. Gradually, through these interactions children learn to regulate their own behavior, a process that lasts well into the mid-20s.^4, 24^ Normal development of these skills then leads to the ability to perform well (on tasks) independently, with no direct need of external rewards.^4^

The interaction between genetic and environmental factors (GxE) also regulates behavioral reward sensitivity, as demonstrated by two earlier studies.^25, 26^ The first reported that parental warmth and stressful life events interacted with a catechol *O*-methyltransferase gene (*COMT*) polymorphism to influence affective decision-making. *COMT* Met allele carriers displayed higher reward sensitivity if they experienced more stressful events, whereas carriers of the Val/Val genotype showed better task performance if they experienced more parental warmth.^25^ The second study observed that adults carrying the dopamine receptor D4 (*DRD4*) 7-repeat allele of the variable number of tandem repeat polymorphism in exon 3 preferred immediate smaller over delayed larger rewards sooner when raised in low socioeconomic status families, but far less when not.^26^ The fact that in both studies genetic variants moderated the sensitivity toward positive and negative environmental influences is in line with the differential susceptibility theory.^27^ This theory states that individuals differ in their susceptibility to environmental experiences (partially) due to genes called ‘plasticity genes', for better and for worse. Thus, individuals carrying specific variants in such genes will not only be most disadvantaged in negative environments but also benefit most from positive environments.^28^ This view extends the commonly applied diathesis-stress or dual-risk models, which focus only on the vulnerability to adverse effects of negative environments, referring to genes involved in GxE as ‘vulnerability genes'.^29, 30^ Support for the differential susceptibility theory comes from various studies in other contexts, although there have also been negative findings see for a recent review Belsky *et al.*^31^

The current study applied the differential susceptibility theory framework to improve our understanding of interindividual differences in reward sensitivity. We aimed to advance prior studies by examining GxE effects on both reward-related behavior and neural activation in children, adolescents and young adults with and without an ADHD diagnosis. As has become clear from the above, reward sensitivity is used in the literature to refer to heterogeneous behavioral measures and neural processes. Here we studied reward sensitivity in the context of behavioral and neural responses to obtain monetary rewards in a modified version of the monetary incentive delay (MID) task.^32^ Functional MRI was used to investigate responses in the ventral striatum and orbitofrontal cortex during reward anticipation and receipt. Using the MID task, our group has shown increased behavioral reward sensitivity and increased activations in the anterior cingulate and anterior frontal cortex during reward anticipation, and in the orbitofrontal cortex and nucleus accumbens during reward receipt in adolescents with ADHD compared with controls.^33^ This is in line with previous studies demonstrating increased behavioral reward sensitivity^34^ and reward-related activations in ADHD,^35, 36, 37, 38^ and studies associating increased striatal activation with impulsivity in healthy subjects, a related concept.^39, 40^ However, findings are not yet consistent as studies in adolescents and (young) adults with ADHD have also reported less striatal activation during reward anticipation compared with controls.^35, 40^ This inconsistency may related to how reward sensitivity was operationalized, as well as to general methodological differences such as small to moderate sample sizes. Nevertheless, our main focus was not on studying differences in reward processing between participants with and without ADHD (as this has been done elsewhere^32^), but rather on investigating GxE effects in an ADHD-enriched sample.

Given its relevance in child development and previous associations with reward sensitivity^19, 21, 22, 23^ we focused on the social environment, which was studied through maternal expressed emotions (EEs) and peer affiliation. Although it has been suggested that the effects of peers are stronger than parental influences in adolescence (see, for example, Harris *et al.*^41^), previous studies have mainly investigated associations between reward sensitivity and parental measures.^19, 21, 22, 25^ As for the candidate genes, we included those variants that have been shown to act as plasticity gene variants in previous studies:^28^ the short allele of the serotonin transporter (*SLC6A4/5-HTT*) *HTTLPR* polymorphism, the 7-repeat allele of the *DRD4* exon 3 variable number of tandem repeat and the 9-repeat allele of the variable number of tandem repeat in the 3′-untranslated region (3′-UTR) of the dopamine transporter gene (*SLC6A3*/ *DAT1*). These genes have been frequently linked to ADHD^42^ and shown to act as plasticity genes in children with and without ADHD.^43^

On the basis of the findings that increased reward sensitivity is related to ADHD and impulsivity,^33, 34, 39, 40, 44^ and adverse environments,^19, 20, 21, 25, 26^ we hypothesized that—if differential susceptibility is applicable—participants with a plasticity variant would show increased reward sensitivity when faced with negative EE or peer affiliation and less reward sensitivity when exposed to positive EE or peer affiliation.

## Materials and methods

### Participants

Participants were selected from a follow-up (2009–2012) of the Dutch part of the International Multicenter ADHD Genetics (IMAGE) study, performed between 2003 and 2006.^45^ At first enrollment in IMAGE, inclusion criteria for children were an age between 5 and 18 years, European Caucasian descent, intelligence quotient ⩾70 and no diagnosis of autism, epilepsy, general learning difficulties, brain disorders or known genetic disorders (such as Fragile X syndrome or Down syndrome). All families were reinvited for a follow-up assessment in NeuroIMAGE at the VU University Amsterdam or Donders Centre for Cognitive Neuroimaging Nijmegen with a mean follow-up period of 5.9 years (s.d.=0.74). A comprehensive assessment protocol was administered, encompassing behavioral questionnaires, a diagnostic interview (for example, of ADHD, oppositional defiance disorder and conduct disorder), several neurocognitive measures from all family members and an extensive MRI scanning protocol in participating children. Participants were asked to withhold the use of psychoactive drugs for 48 h before measurement. To determine ADHD diagnoses at the follow-up measurement, a standardized algorithm was applied to a combination of questionnaires and a semi-structured diagnostic interview (an in-depth description is provided elsewhere^46^). The study was approved by the local ethics committees (Centrale Commissie Mensgebonden Onderzoek), and informed consent was signed by all participants (parents provided consent for participants under 12 years of age).

In the current analyses, participants were included when the reward task was administered and information was available on EE or peer affiliation; *N*=178 participants with ADHD, *N*=44 with subthreshold ADHD (that is, elevated symptoms of ADHD without meeting the full criteria for an ADHD diagnosis) and *N*=221 without ADHD, from *N*=261 families. A flowchart of participant inclusion can be found in Supplementary Figure S1. Sample size depended in particular on the availability of EE and peer affiliation (*N*⩽193 vs *N*⩽429) as EE could only be assessed when the diagnostic interview was administered. This led to an unequal distribution of participants with or without an ADHD diagnosis in the EE vs Peer affiliation selection. Therefore, participant characteristics in Table 1 are displayed separately for EE and peer affiliation.

### Measures

#### Parental expressed emotion

EE was assessed during the semi-structured diagnostic interview, using codings derived from the Camberwell Family Interview.^50^ Only ratings of mothers were used in our study, as the data of fathers were far less complete. Warmth was assessed by the tone of voice, spontaneity, sympathy and/or empathy toward the child (range 0–3). Criticism was assessed by statements, which criticized or found fault with the child based on tone of voice and critical phrases (range 0–4).^51, 52^ Previous studies using similar codings for warmth and criticism have revealed an adequate inter-rater reliability (range 0.71–1.00).^53, 54^

#### Peer affiliation

Peer affiliation refers to the type of friends or peer characteristics a child or adolescent spends time with and was measured with the Friends Inventory.^55^ Participants assessed their peers' behavior on 18 items rated on a 4-point Likert scale (for example, ‘my friends get good grades', ‘my friends break the rules' range 1=‘none of my friends are like that' to 4=‘all of my friends are like that'). Scores were summed to yield either a positive or deviant peer affiliation score (each nine items). Both have demonstrated good internal consistency reliability range (0.88–0.92),^56, 57^ and moderate inter-rater reliability has been reported between self-reports, teacher reports (*α*=0.71 and *r*=0.34–0.43)^55, 58^ and parental reports (*r*=0.38).^56^ Several studies have used peer affiliation as a proxy of the social environment, see for example, Gifford-Smith *et al.*,^59^ Vitaro *et al.*^60^ and Fabes *et al.*^61^

#### ADHD severity

The Dutch Conners Parent Rating scale (CPRS-R:L) was used to assess ADHD severity (that is, the raw scores of scale N: DSM-IV: total).^62^ We used the CPRS-R:L as it was assessed in all participants (regardless of diagnostic status). Moreover, using a continuous measure of ADHD severity allowed us to retain as much information as possible, including the variation of scores among unaffected participants.

#### Reward paradigm

A modified version of the MID task^32, 33^ was used. Participants were instructed to react as quickly as possible to a target (a circle) by pressing a button. A colored square was presented before the target indicating whether a reward could be won or not (green=reward, red=no reward). In the reward condition participants were rewarded with 20 cents if they responded within the presentation time of the target. Trials ended with the presentation of feedback indicating whether the reward was earned or not plus the total amount gained (Supplementary Figure S2). With 25 trials per condition, monetary rewards could add up to a theoretical total of 5 Euros, to be paid at the end of the experiment. However, target presentation time was adapted to the participants' performance (shortened by 20 ms after hits and prolonged 10 ms after misses), resulting in a hit rate of ~33%. This adaptation was done for the reward and non-reward conditions separately to balance the amount of hits on both trial types. As a consequence, hit rate became non-informative as a behavioral measure. The task instruction was followed by a practice trial after which the task began. In order to maximize the design efficiency, the 50 experimental (25 rewarded and 25 non-rewarded) trials were presented in randomized sequence and interleaved with 25 trials without events resulting in a 12-min long experiment.

Behavioral outcome measures of reward sensitivity were reward speeding (mean reaction time (MRT) non-reward-MRT reward) and reward variability (s.d./MRT non-reward-s.d./MRT reward). Neural activation was assessed using the blood-oxygen-level-dependent (BOLD) response during performance on the MID task. After preprocessing of MRI data (details on the image acquisition and preprocessing can be found in the SI) we calculated first-level contrasts for reward anticipation (contrast of the parameter estimates of rewarded cue vs non-rewarded cue; mean number of trials: *M*=22.35, s.d.=2.57) and reward receipt (contrast of parameter estimates of rewarded vs non-rewarded accuracy (hit events vs miss events); mean number of trials: *M*_hits_=7.33, s.d.=1.40; *M*_misses_=14.53, s.d.=2.32). For these two contrasts we extracted the mean BOLD response from two *a priori* defined regions of interest: the ventral striatum (VS) and the ventral medial prefrontal cortex. Both regions of interest are considered core regions of the reward system^8^ and related to ADHD (as described in the introduction). The VS was defined anatomically by segmenting each subject's anatomical MRI scan (FSL FIRST v1.2;^63^ regions labels: 28/56). Because cortical regions cannot be defined anatomically as precise as subcortical regions, the ventral medial prefrontal cortex was defined on the basis of Montreal Neurological Institute (MNI) coordinates derived from a meta-analysis^8^ (0, 52 and −8), with a 10-mm sphere around the coordinates (as in Furukawa *et al.*^35^).

### Genotyping

A description of the genotyping procedure can be found in the Supplementary Information.^64^

### Data analyses

#### Gene–environment correlations

The presence of gene–environment correlations (rGE) could bias potential GxE by providing an alternative explanation for the relationship between environmental measures and genes.^43, 65^ Therefore, Pearson and Spearman correlation analyses (two-sided) were performed to test for rGE between maternal or adolescent candidate plasticity genes and the environmental predictors.

#### Main analyses

Linear mixed model analyses investigated the effects of EE, peer affiliation, genotype and GxE on each reward outcome measure (described above). In addition, we were interested in whether differential susceptibility would be present in general task performance, therefore we focused on the reaction times and variability during reward and non-reward as well. Models were run with and without the interaction term. For both EE and peer affiliation, the positive and negative scales were not sufficiently correlated to create one variable (*r*=−0.50 and −0.16, respectively), therefore maternal warmth, criticism, and positive and deviant peer affiliation were analyzed separately. Likewise, separate models were run for each potential plasticity gene (*DAT1*, *5-HTT* and *DRD4*). Consequently, there were 4 environmental predictors, 3 genes and 10 outcome measures, resulting 3 × 4 × 10 tests.

To correct for familial dependency, as a number of participants belonged to the same families, we estimated a random intercept for family in each model. A random intercept accounts for familial dependency by estimating the correlations between cases within families. Age, gender and collection site were included as potential confounders. All continuous predictors were centered around the mean, and the outcome measures were transformed into normal scores with the use of rank scores via Van der Waerden's formula.^66^

#### Multiple testing correction

A multiple comparisons correction was used, which adjusts for correlated tests based on the effective number (*M*_eff_) of independent comparisons.^67^ The *M*_eff_ was derived from the Eigenvalues of a correlation matrix between the outcome measures adjusted for covariates (age, gender and collection site). In the case of zero correlations between the outcome measures, the *M*_eff_-adjusted *P*-value would be equivalent to a Bonferroni correction. Thus, the *M*_eff_ procedure is particularly suited for correlated comparisons (such as reaction times during reward and non-reward, or neural activation in regions of interest during reward anticipation) and corrects for multiple testing balancing between being overly lenient or conservative. The *M*_eff_ was calculated separately for the behavioral and neural data because of the different nature of the two the types of measures. The effective number of comparisons for both was determined to be 4, and the adjusted *P*-value threshold *P*=0.05/4=0.013.

#### Sensitivity analyses

Sensitivity analyses were performed when significant GxE effects were found (that survived the multiple correction threshold). First, regions of significance (RoS) and simple slope tests were performed with an online application designed for probing interactions in differential susceptibility research (http://www.yourpersonality.net/interaction/, see Roisman *et al.*^68^). Second, to investigate the role of ADHD severity, analyses were rerun including main and interaction effects of ADHD severity. Furthermore, separate sensitivity analyses checked whether significant effects were present in participants while controlling for nonlinear effects of age (age^2^), medication history, intelligence quotient and comorbid oppositional defiance disorder or conduct disorder diagnosis.

#### Code availability

All analyses (except for RoS and simple slope tests) were performed with the Statistical Package for the Social Sciences, version 20.0 (IBM, Armonk, NY, USA). All computer codes used to compute the results are available on www.neuroimage.nl.

## Results

A significant rGE was found between adolescent *DRD4* genotype and deviant peer affiliation (*r*=0.11, *P*=0.028; Supplementary Table S1). Furthermore, maternal *DAT1* was negatively correlated with maternal warmth (*r*=−0.18, *P*=0.015), and maternal *5-HTT* associated with deviant peer affiliation and maternal warmth (*r*=0.10, *P*=0.045; *r*=−0.20, *P*=0.005, respectively). Significant rGEs, however, were relatively small, and are unlikely to have biased possible GxE interactions. In describing the outcomes of the mixed model analyses we restricted ourselves to the results that survived correction for multiple testing. Nominally significant effects can be found in Supplementary Table S2–S5.

### Reward speeding

**Figure 1:**
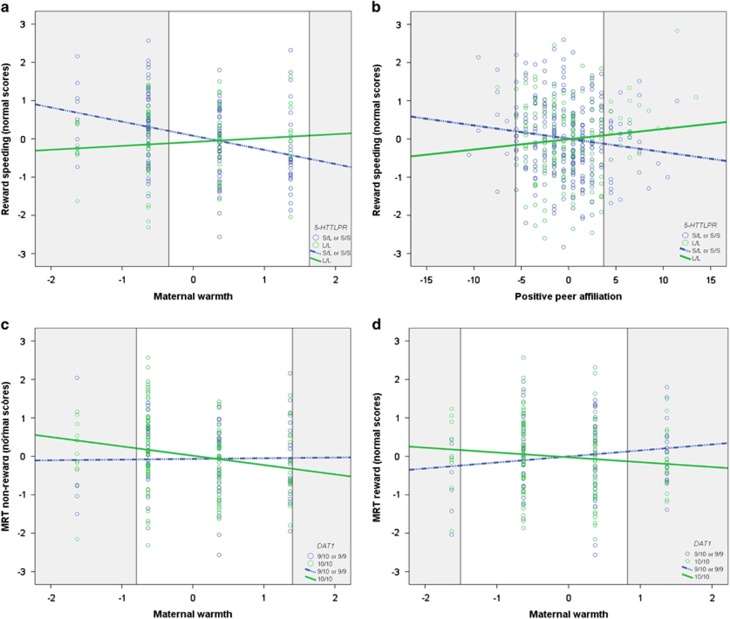
(**a**) Interaction between *5-HTT* and maternal warmth on reward speeding (*B*=−0.45, *P*=0.005; normal score (0)=27.71 ms). The shaded areas indicate the regions of significance (RoS), lower threshold *X*=−0.34; upper threshold *X*= 1.64. (**b**) Interaction between *5-HTT* and positive peer affiliation on reward speeding (*B*= −0.07, *P*=0.012; normal score (0)=25.52 ms). The shaded areas indicate the RoS, lower threshold *X*=−5.61; upper threshold *X*= 3.71. (**c**) Interaction between *DAT1* and maternal warmth on the mean reaction time during non-reward (*B*=0.40, *P*=0.012; normal score (0)=324.90 ms). The shaded areas indicate the RoS, lower threshold *X*=−0.80; upper threshold *X*=1.40. (**d**) Interaction between *DAT1* and maternal warmth on the mean reaction time during reward (*B*=0.41, *P*=0.013; normal score (0)=296.31 ms). The shaded areas indicate the RoS, lower threshold *X*=−1.51; upper threshold *X*=0.82. Values in the RoS are significant. MRT, mean reaction time.

Our linear mixed model showed that both maternal warmth and criticism were significantly associated with adolescent reward speeding (*B*_warmth_=−0.19, *P*=0.013; *B*_criticism_=0.20, *P*=0.008; Supplementary Table S2). For maternal warmth this effect was moderated by *5-HTT* genotype (*B*=−0.45, *P*=0.005; Supplementary Table S3). A similar GxE interaction was found between *5-HTT* and positive peer affiliation (*B*=−0.07, *P*=0.012). As can be seen in Figures 1a and b, participants with the *HTTLPR* short allele showed a significant negative association between reward speeding and maternal warmth and positive peer affiliation. Simple slope analyses revealed both slopes were significant (*p*_positive_=0.049, *p*_warmth_<0.001), whereas slopes for participants with the *HTTLPR* L/L genotype were not (*p*_positive_=0.994 and *p*_warmth_=0.558). However, inspection of the RoS with respect to the environmental predictors revealed the difference between the two genotypes was not significant for high warmth (that is, no values fell above the upper RoS threshold *X*=1.64). Thus, *HTTLPR* short-allele carriers showed the most reward speeding when exposed to low maternal warmth (RoS threshold *X*=−0.34) or low positive peer affiliation (RoS threshold *X*=−5.61), but the least when exposed to high positive peer affiliation (RoS threshold *X*=3.71) when compared with adolescents with the *HTTLPR* L/L genotype.

Subsequent analyses of the reaction times in each condition separately (reward vs non-reward) revealed a significant interaction between *DAT1* and maternal warmth. Opposite to our predictions, the MRT was negatively associated with maternal warmth in participants with the *DAT1* 10/10 genotype, regardless of reward condition, see Figures 1c and d (*B*_reward_=0.41, *P*=0.013; *B*_non-reward_=0.40, *P*=0.012). Simple slope analyses revealed the slopes were significant for participants with the *DAT1* 10/10 genotype (*p*_reward_=0.044, *p*_non-reward_=0.001), but not for 9-repeat carriers (*p*_reward_=0.103, *p*_non-reward_=0.567). Hence, *DAT1* 10/10 homozygotes had the longest reaction times when exposed to low warmth, but the shortest when exposed to high warmth, compared with *DAT1* 9-repeat carriers. However, no values of the non-reward reaction times fell within the upper RoS threshold for maternal warmth (*X*=1.40). Therefore, here *DAT1* 10-repeat homozygotes only differed significantly from each other when exposed to low maternal warmth (RoS threshold *X*=−0.80). For reward reaction times, values fell within both the lower (*X*=−1.51) and upper RoS threshold (*X*=0.82).

### Reward variability

Analyses of reward variability showed no effects that survived correction for multiple testing (all *P*-values >0.018; Supplementary Table S2). Looking at the conditions separately, no effects were present in the reward or non-rewarded condition either (all *P*-values >0.030).

### Neural activation

**Figure 2:**
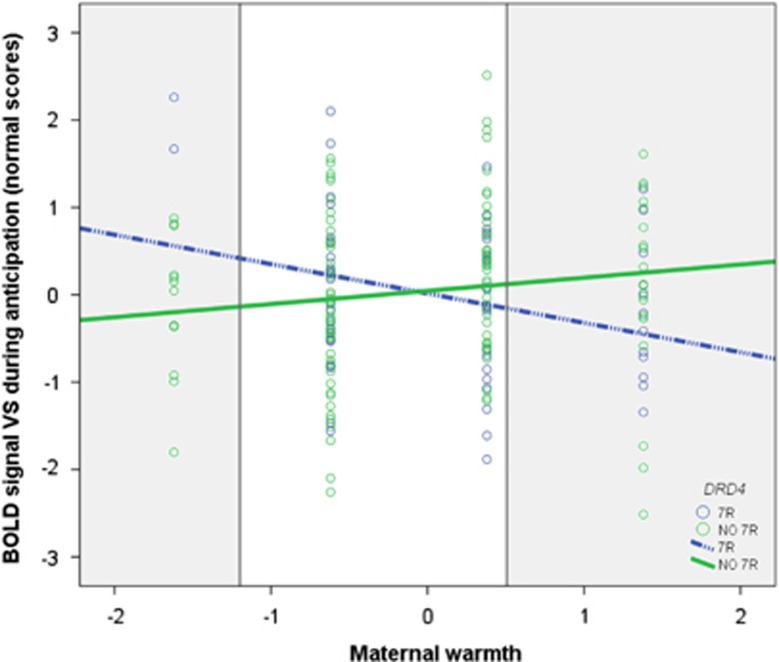
Interaction between *DRD4* and maternal warmth on the activation in the ventral striatum during reward anticipation (*B*=−0.55, *P*=0.004; normal score (0)=blood-oxygen-level-dependent (BOLD) signal change 197.42). The shaded areas indicate the regions of significance (RoS), lower threshold *X*=−1.20; upper threshold *X*= 0.51. Values in the RoS are significant.

A significant interaction between *DRD4* and maternal warmth was found for VS activation during reward anticipation, shown in Figure 2 (*B*=−0.55, *P*=0.004; Supplementary Table S5). Simple slope analyses revealed only the slope of *DRD4* 7-repeat carriers was significant (carriers: *P*=0.014; non-carriers: *P*=0.140). Adolescents with the 7-repeat allele showed the highest activation when exposed to low maternal warmth (RoS threshold *X*=−1.20), but lowest when exposed to high warmth (RoS threshold *X*=0.51), compared with those without the 7-repeat. Furthermore, separate main effect analyses indicated that maternal criticism was positively associated with the VS BOLD response during reward receipt (*B*=0.21, *P*=0.009; Supplementary Table S4). No interactions were found during reward receipt in the VS or for the ventral medial prefrontal cortex activation (all *P*-values >0.109).

### Sensitivity analyses

Sensitivity analyses were performed to check whether the above-described significant GxE interactions were affected by ADHD severity, as measured by the CPRS. These revealed no significant three-way interactions (all *P*-values >0.175). Moreover, including ADHD severity as a main effect did not change significant GxE effects. Finally, accounting for nonlinear age effects, intelligence quotient, oppositional defiance disorder, conduct disorder or medication history by rerunning the analyses for significant GxE effects while separately including these measures in the model did not affect GxE interactions.

## Discussion

We found evidence for differential genetic susceptibility toward positive social environments for behavioral-related and striatal sensitivity to rewards in a large sample of adolescents, independent of ADHD severity. Up to now, authors have speculated about the role of the brain when investigating GxE effects on reward sensitivity. We believe we showed here for the first time that *DRD4* genotypes moderate the association between warmth and neural responses to the anticipation of rewards in the VS.

Several explanations have been outlined to understand the relationship between reward-seeking behavior in daily life, as observed in adolescence, and neural activation during reward processing in imaging paradigms.^11^ Current evidence suggests that a hyper-responsive neural reward system predisposes to greater reward seeking, whereby increased dopaminergic release in response to rewarding events strengthens reward-related behavior through dopamine-based learning processes.^11, 12, 13^ In agreement with this perspective, the genetic moderation of both behavioral and neural responsiveness found in this paper could be explained by altered transcriptional activity, which affects the amount of dopamine released. For example, the *DRD4* 7-repeat polymorphism is associated with decreased postsynaptic inhibition of dopamine, which in turn leads to increased levels of dopamine.^69^ In addition, animal studies have demonstrated an association between maternal deprivation and increased dopamine levels.^70^ Further, studies in humans have revealed protective effects of positive parenting,^19, 21^ as well as detrimental effects of low warmth on behavioral and neural measures of reward sensitivity. Differential effects toward the environment then might be caused by exacerbation of dopamine increase in negative environments, but compensation when exposed to positive influences. This idea is supported by our finding of *DRD4* 7-repeat carriers showing the most striatal activation during reward anticipation when exposed to low maternal warmth, but the least when exposed to high levels of warmth.

Similar processes might occur for the *5-HTT* gene, as participants carrying the *HTTLPR* short allele showed the least reward speeding when exposed to high positive peer affiliation, but the most when faced with low positive peer affiliation or low maternal warmth. Similar to the *DRD4* 7-repeat variant, decreased transcriptional activity has been associated with the *HTTLPR* short allele, resulting in an excess of serotonin levels.^71^ Besides dopamine, serotonin is also relevant for reward processing, and it is suggested that the interaction between dopamine and serotonin controls the behavioral response to rewards.^72^

For *DAT1*, in contrast to what we expected, we found *DAT1* 10/10 homozygotes displayed a similar differential pattern toward warmth for general task performance in both rewarded and non-rewarded conditions. On the basis of a previous GxE study in children with ADHD^51^ we had hypothesized that the 9-repeat would be the plasticity variant. However, evidence for the 10-repeat as candidate plasticity variant has been found in a community study,^73^ although neither study focused on reward sensitivity. Similar mixed results have been reported as to whether the 9- or 10-repeat shows increased or decreased expression.^74^ Finally, it is important to note that other variants and epigenetic factors not included in this study may influence the functional levels of the genes we investigated.^75, 76^ Considering how much is still unknown about the exact workings of dopamine and serotonin variation as a consequence of gene variants, especially in relation to environmental effects, more research is needed before we can truly state which and how gene variants enhance susceptibility.

Taken together, our findings partially support the differential susceptibility theory. Yet, this theory states individuals carrying plasticity alleles are sensitive to both positive and negative environments,^43^ while our results almost exclusively involved positive environments. However, besides less reward sensitivity when exposed to positive environments, the present study revealed increased reward sensitivity when faced with low positive environments as well. Although seemingly not the best way to operationalize an adverse environment, the absence of a positive environment is often associated with negative effects in child development, for example, Newman *et al.*^77^ and Yap *et al.*^89^ Therefore, viewing low warmth or low positive peer affiliation as adverse experiences seems valid, thereby placing the results in line with the differential susceptibility theory.

Two GxE interactions were found that do not fit the criteria of differential susceptibility: the interactions of *5-HTT* and *DAT1* with warmth on reward speeding and non-rewarded reactions times, respectively. Here carriers of the candidate susceptibility variants only differed from non-carriers when exposed to low maternal warmth. When viewing low warmth as a form of adversity (as argued above), these findings are more in line with the diathesis-stress model.^29, 30^ This theory states that genes moderate a person's vulnerability to adverse effects only, while making no differences in positive environments.^43^ Thus, focusing on the same candidate plasticity gene and reward outcome measure (*5-HTT* and reward speeding), but different environmental measures (warmth vs positive peer affiliation), or focusing on the same gene and environmental predictor (*DAT1* and warmth), but different outcome measures (rewarded vs non-rewarded MRT) led to the support for either differential susceptibility or diathesis-stress. These results demonstrate the complexity of how and in which situations individuals differ in their susceptibility toward environmental experiences.

The absence of significant interactions with the negative social environment in this study could indicate that positive social environments are more important for reward sensitivity. Indeed, the positive social environment has a key role in the development of reward learning (as described in Introduction). Our findings are in line with the idea that positive social influences promote optimal reward learning, and more so for adolescents with particular genotypes. We did, however, find main effects of criticism on reward receipt activation and reward speeding, as well as nominally significant interactions effects with both negative environmental measures. Therefore, investigation of both positive and negative effects in larger samples and from different populations is warranted before further conclusions as to which social environment has a stronger role in the development of reward learning.

This study had a number of strengths and limitations. Strengths were the use of a well-characterized sample, inclusion of both positive and negative environments, with both parental- and peer influences assessed, and the analysis of both behavioral and neural measures of reward processing. A limitation is the cross-sectional study design; longitudinal studies are needed to establish a direction of causality. Establishing the direction of effects is particularly difficult when focusing on parental and peer factors. Indeed, both maternal EE and peer affiliations have not only been suggested to influence child behavior, but in turn be influenced by child behavior as well.^78, 79, 80, 81, 82, 83, 84, 85^ Another possible limitation is that peer affiliation was measured by self-report and therefore reflects perception rather than an objective measure. Direct observations of peer affiliations would have eliminated the possible bias of self-observation and may have been the ideal measure. However, this is very difficult, if not impossible, to achieve in adolescence and young adulthood and was not feasible within our study as it was not solely aimed at investigating peer affiliations. Furthermore, not all participants included had an EE measurement, as it was assessed when a full diagnostic interview was administered. This led to loss of power, unequal numbers and an unequal distribution of ADHD and controls in the EE vs peer affiliation analyses. Nevertheless, the GxE effect on reward speeding was present in both social environments and most interaction effects were found for EE only, suggesting that it is a powerful moderator. Moreover, sensitivity analyses revealed no effect of ADHD severity on significant GxE interactions. Previous power calculations with a sample size of ~350 indicated that we had adequate power to detect GxE effects with an explained variance of 3–5% or higher,^86^ but this amount of variance is considered to be quite large in the GxE literature^87^ and smaller but still relevant effects may go undetected. We therefore emphasize the need of replication studies. Finally, as we used a modified version of the MID task with a lower hit rate compared with the original version of the task (33% vs 66%), it could be suggested that this might have led to participants finding the task too difficult and to feelings of frustration. However, the value of rewards has been suggested to depend on the context.^88^ Consequently, the lower hit rate would only have been experienced as frustrating when participants had been able to compare it with higher hit rates (see also von Rhein *et al.*^33^). Still, here too replication studies are a necessity.

In conclusion, these results indicate GxE interplay is relevant for an improved understanding of interindividual differences in behavioral and neural measures of reward sensitivity and general task performance. Our findings may ultimately also have implications for clinical settings, as targeting parents or peers of at-risk adolescents could be particularly helpful for carriers of the *HTTLPR* short allele and the *DRD4* 7-repeat. Importantly, our results were not modified by ADHD severity. This suggests that the effects of genes, social environment and their interplay contribute in a general way to interindividual differences in striatal responses during reward anticipation, reward speeding and general task performance, and are not specific for ADHD. Considering the new research questions and novel findings, more research on GxE interactions and reward sensitivity is needed, in particular replication of our findings in independent large data sets with additional types of positive and negative social environments.

## Figures and Tables

**Table 1:** Participant characteristics

	*Expressed emotion selection*			*Peer affiliation selection*		
	N	M	*s.d.*	N	M	*s.d.*
Number of families	150			261		
						
ADHD diagnosis	153	79%		166	39%	
Inattentive subtype	68	35%		74	17%	
Hyperactive–impulsive subtype	18	9%		25	6%	
Combined subtype	67	35%		67	16%	
Subthreshold ADHD	19	10%		43	10%	
Unaffected	21	11%		220	51%	
ADHD severity (CPRS)	191	20.94	12.18	418	12.08	12.25
ODD diagnosis	46	24%		50	12%	
CD diagnosis	11	6%		11	6%	
History of stimulant use	136	70%		145	34%	
Male	127	66%		237	55%	
Collection site (Amsterdam)	80	42%		216	50%	
Age	193	17.15	3.24	429	17.48	3.52
Estimated IQ	193	97.54	14.85	426	101.62	12.25
Maternal warmth/positive peer affiliation	193	1.64	0.89	429	22.52	3.58
Maternal criticism/deviant peer affiliation	193	1.65	0.92	429	15.01	4.44
MRT reward condition (ms)	193	298.81	39.44	429	298.25	36.73
MRT non-reward condition (ms)	193	332.05	50.01	429	329.48	47.62
Variability reward condition (ms)	193	0.21	0.14	429	0.19	0.11
Variability non-reward condition (ms)	193	0.25	0.15	429	0.24	0.15
						
*Bold response reward anticipation*						
VS	167	196.90	841.49	375	267.14	828.11
vmPFC	167	−508.21	1882.21	375	−412.53	2132.62
						
*Bold response reward receipt*						
VS	167	604.36	1542.24	375	408.06	1540.83
vmPFC	167	1722.58	3714.42	375	1498.62	4225.33
						
*DAT1*	186			407		
9-repeat present	62^a^	33%		150^b^	37%	
9-repeat absent^c^	124	67%		257	63%	
						
*5-HTT*	190			416		
Short allele present	123^d^	65%		269^e^	65%	
Short allele absent	67	35%		147	35%	
						
*DRD4*	190			417		
7-repeat present	64	34%		143	34%	
7-repeat absent	126	66%		274	66%	

Abbreviations: ADHD, attention-deficit/hyperactivity disorder; CD, conduct disorder; CPRS, Conners Parent Raring Scale; IQ, intelligence quotient; MRT, mean reaction time; ODD, oppositional defiance disorder; vmPFC, ventral medial prefrontal cortex; VS, ventral striatum.

ODD and CD diagnoses were based on Kiddie-Schedule for Affective Disorders and Schizophrenia (K-SADS) structured psychiatric interviews.^47^

Estimated IQ was based on two subtests of the Wechsler Intelligence Scale for Children (WISC)/ Wechsler Adult Intelligence Scale (WAIS-III): Vocabulary and Block Design.^48, 49^

a *N*=12 (7%) with two 9-repeats.

b *N*=20 (5%) with two 9-repeats.

c 10/10 genotype.

d *N*=28 (15%) with two short alleles.

e *N*=59 (14%) with two short alleles.
