# Supplementary material for: Adolescent behavioral and neural reward sensitivity: a test of the differential susceptibility theory
Source: Transl Psychiatry. 2016 Apr 5;6(4):e771–. doi: 10.1038/tp.2016.37 (PMC4872395; doi:10.1038/tp.2016.37)
Supplement: Supplementary Information [file tp201637x1.doc]

**Supplemental Information**

**Participant Inclusion**

Figure S1 shows the exact numbers for inclusion and details on exclusion criteria. The reward task was administered to 571 participants, of which 453 had correct behavioral and 395 correct neural data. Main reason of exclusion was an insufficient number of trials per event type (less than 5, N=83). Sample size depended further in particular on the availability of expressed emotion (EE) and peer affiliation. EE was assessed only when the diagnostic interview was administered. Final sample sizes depended on the availability of genotypes.

**
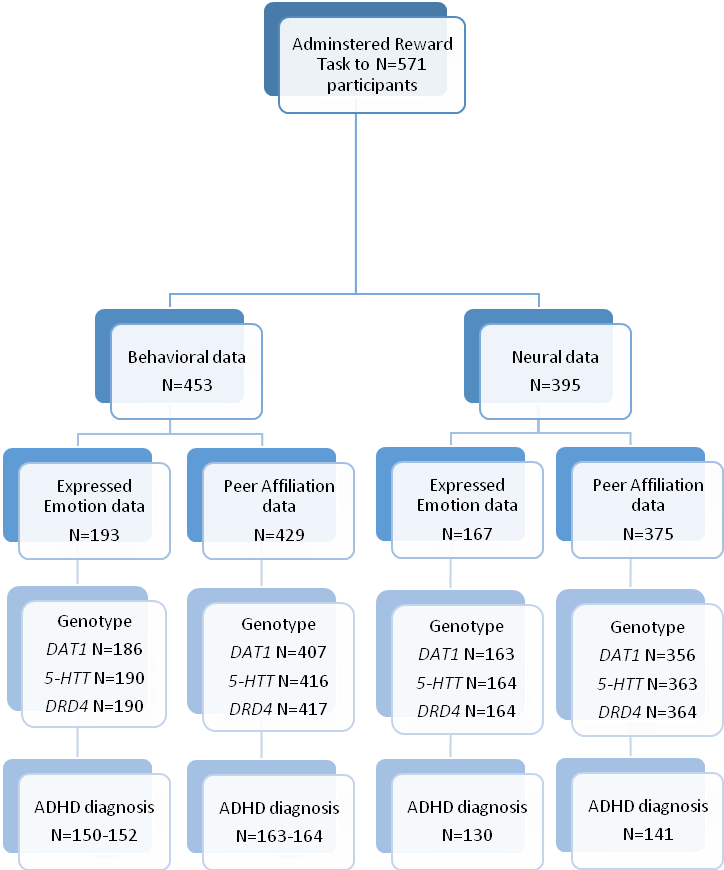
**

*Participants excluded (N=118)*

Did not stop medication use N=7

Drug use 24h prior testing N=1

Autism diagnosis N=1

Claustrophobia N=8

Technical problems N=6

Incomplete data N=10

Unreliable data N=2

Insufficient trials per event type (<5) N=83

*Additional exclusion (N=58)*

Technical problems N=18

Excessive movement N=6

Scientific or clinically relevant findings N=8

Insufficient clean data N=26

*Figure S1*. Flowchart of participant inclusion for data on the reward task, expressed emotion, peer affiliation and genotype. Final numbers under ‘genotype’ depict the numbers used in analyses of gene-environment interactions.


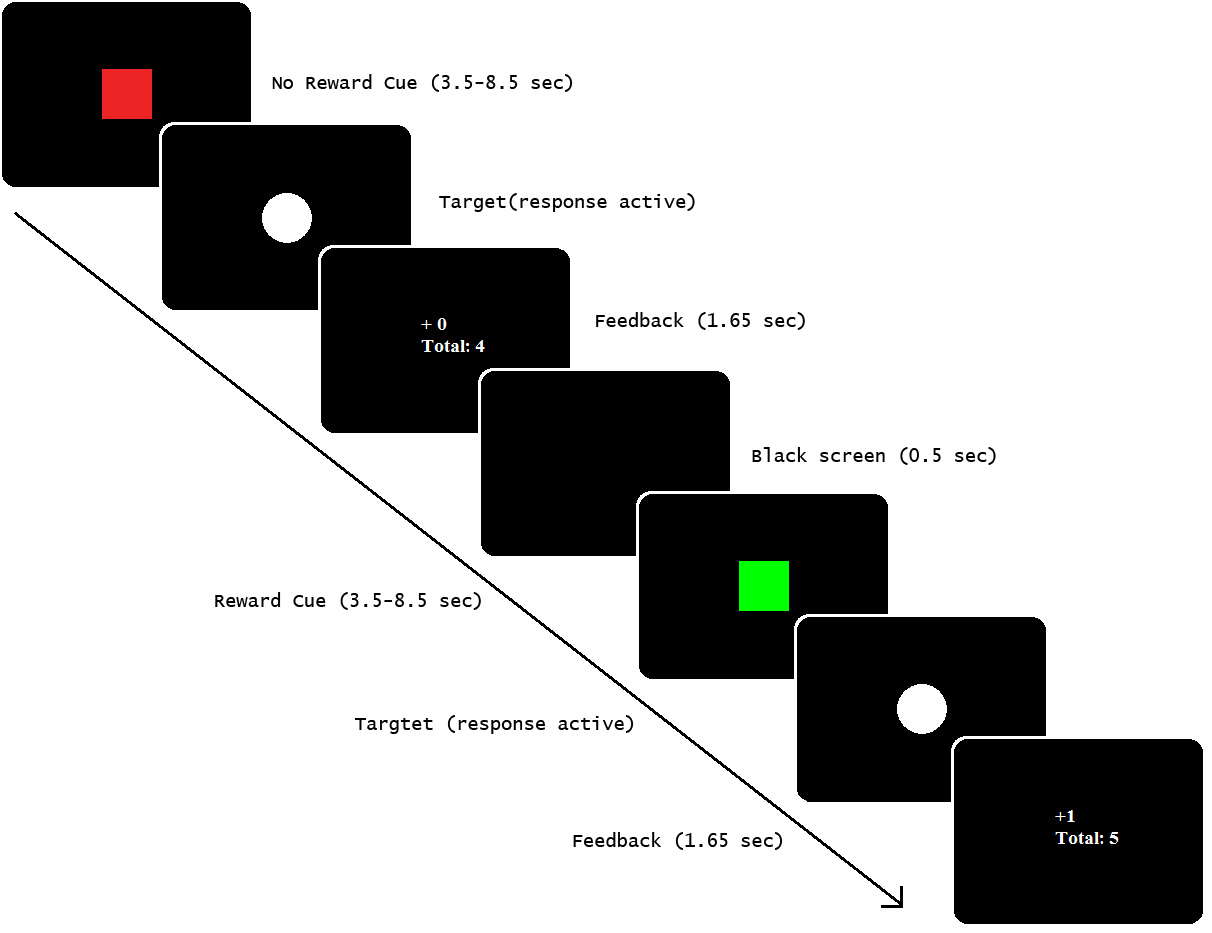


**+ 0.20**

**Total: 1.00**

**+ 0**

**Total: 0.80**

**Target**

**Target**

*Figure S2*. Schematic overview of the Monetary Incentive Delay task

**fMRI Acquisition**

Imaging was conducted at two locations (VU University Amsterdam and Donders Center for Cognitive Neuroimaging Nijmegen) using two comparable 1.5 Tesla MRI scanners (Avanto / Sonata Siemens, Munich, Germany), identical product 8-channel head coils and closely matched scan protocols. Whole brain functional imaging was performed using a gradient-echo echo-planar scanning (EPI) sequence (37 axial slices, repetition time = 2340 ms, echo time = 40 ms, voxel size = 3.5 x 3.5 x 3.0 mm, interslice gap = 0.5 mm, field of view = 224 mm, flip angle =90°). Before acquisition of functional images, a high-resolution T1-weighted MP-RAGE anatomical scan was obtained (176 sagittal slices, repetition time = 2730 ms, echo time = 2.95 ms, inversion time = 1000 ms, flip angle = 7°, voxel size = 1.0 x 1.0 x 1.0 mm, field of view = 256 mm). The MID task was part of a longer scanning session that included other fMRI and DTI scans. The order of fMRI scans was randomized across participants. BOLD time series data were preprocessed and analyzed using SPM8 (Wellcome Department of Cognitive Neurology, London).

**fMRI Preprocessing**

All echo planar images (EPIs) were analyzed using FSL (FSL version 5, . First, the first 5 volumes were discarded to allow for T1 equilibrium. Then, all remaining EPIs were spatially realigned to the middle volume to correct for head movement (FSL MCFLIRT ). After performing nuisance regression using the extended realignment parameters estimated during realignment and the extracted time courses of regions containing white matter and cerebral-spinal fluid, images were spatially smoothed (FWHM=6mm), and a temporal highpass filter was applied (cut-off=0.01Hz). Each subject’s EPI images were spatially co-registered to its corresponding anatomical image using FSL’s FLIRT boundary-based registration algorithm and normalized to MNI152 standard space after performing first level statistics. To avoid registration bias when registering adolescent brains to the adult-based MNI152 template we used a custom-built study template. This template was generated by averaging across all T1-scans of participants in the NeuroIMAGE study (N = 787), with a resolution of 2 x 2 x 2 mm after non-linear transformation to MNI152 space using FSL FNIRT ([www.fmrib.ox.ac.uk/fsl](http://www.fmrib.ox.ac.uk/fsl)). Subsequently we recalculated each participant’s non-linear warp-field from its T1 to the custom template (FSL FNIRT).

**fMRI Analysis**

Statistical parametric maps were estimated for each participant with a general linear model (GLM; FSL FEAT). First-level regressors included six regressors of interest (onset times of rewarded and non-rewarded cues, hits, and misses, each with a duration of 0 seconds) and 6 regressors of no interest. Regressors of no interest comprised a) onsets of rewarded and non-rewarded targets, b) cue, target, and outcome onsets of error events, and c) a motion regressor. Error events comprised events of trials with incorrect responses (i.e. premature responses (RT < 100 ms), too many (>1) or too early (i.e. before target onset) button presses or no response at all). The motion regressor was inserted to control for possible movement artifacts as described by Keulers and colleagues . Head movements from one image to the next exceeding a threshold of 0.5 mm in either the x, y or z direction were considered movement artifacts. Onset of this error event was set to 8 seconds before the movement and all events of interest within this 8 seconds interval were discarded. All regressors and their temporal derivatives were convolved with a canonical hemodynamic response function (HRF) and fitted in the GLM. Finally, the estimated beta maps for each subject were normalized to a common space (MNI152).

**Genotyping**

For the IMAGE sample (parents and children), DNA was extracted from blood samples or immortalized cell lines at Rutgers University Cell and DNA Repository, New Jersey, USA. The genetic variants in *DAT1,* 5-*HTT,* and *DRD4*, were genotyped by the IMAGE consortium . Additional NeuroIMAGE samples were collected in the form of a saliva sample using Oragene kits (DNA-Genotek; see [www.neuroimage.nl](http://www.neuroimage.nl/)). Genotyping of addition samples was performed as described in . No deviations from Hardy-Weinberg Equilibrium were found (*DAT1* *p*=.78, 5-*HTT* *p*=.13, *DRD4* *p*=.15). For each gene, participants were divided into groups based on the presence or absence of the candidate plasticity alleles (i.e. the 9-repeat of the *DAT1* 3’UTR VNTR, the short allele of *HTTLPR*, the 7-repeat of the *DRD4* exon 3 VNTR). Data on DNA sequences are available on [www.neuroimage.nl](http://www.neuroimage.nl/).

Table S1

*Correlations between maternal or adolescent’s genotype and maternal expressed emotions and peer affiliation*

|  | Positive peer affiliation | Deviant peer affiliation | Maternal warmth | Maternal criticism |
| --- | --- | --- | --- | --- |
| Adolescent’s genotype |  |  |  |  |
| *DAT1*^a^ | -.04 | .06 | -.13 | .08 |
| *5-HTT*^b^ | .00 | .05 | -.11 | .07 |
| *DRD4*^c^ | .02 | .11* | -.07 | .14 |
|  |  |  |  |  |
| Maternal genotype |  |  |  |  |
| *DAT1*^a^ | -.07 | .06 | -.18* | .07 |
| *5-HTT*^b^ | .00 | .10* | -.20** | .09 |
| *DRD4*^c^ | .06 | -.01 | -.11 | .04 |

*Note*: Pearson correlation analyses were performed for the EE measures and Spearman correlations for peer affiliation. ^a^ 9-repeat allele present or absent; ^b^ short allele present or absent; ^c^ 7-repeat allele present or absent short allele present or absent.

* Significant at *p* ≤ .05

** Significant at *p* ≤ .01

Table S2

*Mixed model analyses testing the separate main effects of maternal expressed emotions, peer affiliation* and plasticity genes on behavioral measures of reward sensitivity

|  |  | Reward speeding | | | RT  reward | | | RT  non-reward | | | Reward  variability | | | Variability  reward | | | Variability  non-reward | | |
| --- | --- | --- | --- | --- | --- | --- | --- | --- | --- | --- | --- | --- | --- | --- | --- | --- | --- | --- | --- |
|  |  | *B* | *SE* | *p* | *B* | *SE* | *p* | *B* | *SE* | *p* | *B* | *SE* | *p* | *B* | *SE* | *p* | *B* | *SE* | *p* |
| Main EE effects | |  |  |  |  |  |  |  |  |  |  |  |  |  |  |  |  |  |  |
| 1 | Warmth | **-.19** | **.08** | **.013** | -.05 | .08 | .502 | ***-.17*** | ***.07*** | ***.023*** | -.12 | .08 | .116 | -.01 | .08 | .905 | -.14 | .08 | .070 |
| 2 | Criticism | **.20** | **.07** | **.008** | -.10 | .07 | .188 | .07 | .07 | .370 | .13 | .08 | .080 | .03 | .08 | .716 | ***.15*** | ***.08*** | ***.046*** |
|  |  |  |  |  |  |  |  |  |  |  |  |  |  |  |  |  |  |  |  |
| Main PA effects | |  |  |  |  |  |  |  |  |  |  |  |  |  |  |  |  |  |  |
| 1 | Positive PA | -.01 | .01 | .500 | .00 | .01 | .765 | -.01 | .01 | .602 | -.01 | .01 | .421 | -.02 | .01 | .227 | -.02 | .01 | .237 |
| 2 | Deviant PA | .01 | .01 | .536 | ***-.03*** | ***.01*** | ***.017*** | -.01 | .01 | .197 | .01 | .01 | .504 | .02 | .01 | .190 | ***.02*** | ***.01*** | ***.045*** |
|  |  |  |  |  |  |  |  |  |  |  |  |  |  |  |  |  |  |  |  |
| Main gene effects | |  |  |  |  |  |  |  |  |  |  |  |  |  |  |  |  |  |  |
| 1 | *DAT1*^a^ | .08 | .10 | .403 | .03 | .10 | .793 | .09 | .10 | .382 | -.02 | .10 | .837 | -.07 | .10 | .488 | -.08 | .10 | .443 |
| 2 | *5-HTT*^b^ | -.03 | .10 | .780 | .05 | .10 | .646 | .05 | .10 | .631 | -.10 | .10 | .316 | .12 | .10 | .219 | .04 | .10 | .701 |
| 3 | *DRD4*^c^ | .06 | .10 | .581 | .08 | .10 | .457 | .11 | .10 | .270 | -.06 | .10 | .535 | .07 | .10 | .518 | .01 | .10 | .915 |

*Note:* ^a^ Reference group: 9-repeat absent; ^b^ reference group: short allele absent; ^c^ reference group: 7-repeat absent. PA=peer affiliation. Findings in bold are significant after correction for multiple testing (*p* < .013), findings in bold and italic are nominally significant (i.e. not significant after correction for multiple testing; *p* ≤ .05). All analyses were corrected for age, gender and collection site.

Table S3

*Mixed model analyses testing interaction effects between plasticity genes and maternal expressed emotion or peer affiliation on behavioral measures of reward sensitivity*

|  |  | Reward speeding | | | RT  reward | | | RT  non-reward | | | Reward  variability | | | Variability  reward | | | Variability  non-reward | | |
| --- | --- | --- | --- | --- | --- | --- | --- | --- | --- | --- | --- | --- | --- | --- | --- | --- | --- | --- | --- |
|  |  |
|  |  | *B* | *SE* | *p* | *B* | *SE* | *p* | *B* | *SE* | *p* | *B* | *SE* | *p* | *B* | *SE* | *p* | *B* | *SE* | *p* |
|  |  |  |  |  |  |  |  |  |  |  |  |  |  |  |  |  |  |  |  |
| 1 | Warmth | **-.26** | **.10** | **.009** | ***-.20*** | ***.10*** | ***.044*** | **-.33** | **.10** | **.001** | ***-.23*** | ***.10*** | ***.026*** | .05 | .10 | .640 | -.16 | .10 | .121 |
|  | *DAT1*^a^ | -.09 | .15 | .548 | .04 | .15 | .782 | -.06 | .14 | .657 | -.14 | .15 | .374 | -.01 | .15 | .944 | -.20 | .15 | .192 |
|  | *DAT1*^a^ *Warmth | .11 | .17 | .525 | **.41** | **.16** | **.013** | **.40** | **.16** | **.012** | .27 | .17 | .116 | -.06 | .17 | .731 | .10 | .17 | .544 |
| 2 | Warmth | .08 | .13 | .539 | -.07 | .13 | .608 | .00 | .12 | .975 | .04 | .13 | .763 | -.09 | .13 | .505 | -.06 | .13 | .665 |
|  | *5-HTT*^b^ | .14 | .14 | .329 | .06 | .14 | .672 | .15 | .14 | .265 | .08 | .15 | .591 | .09 | .15 | .551 | .21 | .15 | .147 |
|  | *5-HTT*^b^*Warmth | **-.45** | **.16** | **.005** | .03 | .16 | .854 | -.27 | .15 | .079 | -.28 | .17 | .087 | .15 | .17 | .372 | -.15 | .17 | .366 |
| 3 | Warmth | -.14 | .09 | .137 | -.09 | .09 | .311 | -.16 | .09 | .070 | -.06 | .09 | .501 | .00 | .09 | .959 | -.08 | .10 | .422 |
|  | *DRD4*^c^ | -.07 | .15 | .634 | .06 | .15 | .659 | .03 | .14 | .843 | -.10 | .15 | .510 | .00 | .15 | .983 | -.06 | .15 | .684 |
|  | *DRD4^c^**Warmth | -.21 | .17 | .225 | .16 | .17 | .349 | -.03 | .17 | .877 | -.24 | .17 | .179 | .05 | .18 | .760 | -.23 | .18 | .197 |
|  |  |  |  |  |  |  |  |  |  |  |  |  |  |  |  |  |  |  |  |
| 1 | Criticism | .13 | .09 | .150 | .01 | .09 | .914 | .09 | .09 | .333 | .15 | .10 | .119 | -.02 | .10 | .825 | .14 | .10 | .146 |
|  | *DAT1*^a^ | -.10 | .15 | .507 | .03 | .15 | .849 | -.08 | .14 | .599 | -.13 | .15 | .391 | .01 | .15 | .968 | -.19 | .15 | .199 |
|  | *DAT1*^a^*Criticism | .17 | .17 | .312 | -.29 | .17 | .091 | -.07 | .17 | .679 | -.04 | .18 | .815 | .19 | .17 | .269 | .08 | .17 | .653 |
| 2 | Criticism | .08 | .11 | .490 | -.02 | .11 | .888 | .03 | .11 | .791 | .02 | .11 | .865 | .16 | .11 | .170 | .13 | .11 | .247 |
|  | *5-HTT*^b^ | .14 | .14 | .338 | .04 | .14 | .758 | .14 | .14 | .321 | .08 | .15 | .604 | .09 | .15 | .551 | .20 | .15 | .168 |
|  | *5-HTT^b^**Criticism | .23 | .15 | .121 | -.14 | .15 | .358 | .08 | .15 | .602 | .21 | .15 | .176 | -.23 | .15 | .136 | .04 | .15 | .809 |
| 3 | Criticism | ***.18*** | ***.09*** | ***.046*** | -.08 | .09 | .365 | .07 | .09 | .450 | .15 | .09 | .089 | .02 | .09 | .838 | .17 | .09 | .069 |
|  | *DRD4*^c^ | -.05 | .15 | .717 | .05 | .15 | .753 | .02 | .15 | .866 | -.11 | .15 | .462 | .01 | .15 | .940 | -.07 | .15 | .661 |
|  | *DRD4*^c^*Criticism | .08 | .17 | .617 | -.04 | .17 | .809 | .02 | .16 | .918 | -.10 | .17 | .576 | .04 | .17 | .838 | -.05 | .17 | .753 |
|  |  |  |  |  |  |  |  |  |  |  |  |  |  |  |  |  |  |  |  |
| 1 | Positive PA | -.03 | .02 | .053 | .00 | .02 | .852 | -.02 | .02 | .127 | -.02 | .02 | .153 | -.01 | .02 | .671 | -.02 | .02 | .177 |
|  | *DAT1*^a^ | .08 | .10 | .411 | .03 | .10 | .752 | .09 | .10 | .369 | -.02 | .10 | .816 | -.05 | .10 | .594 | -.07 | .10 | .457 |
|  | *DAT1*^a^ *Positive PA | ***.07*** | ***.03*** | ***.013*** | -.01 | .03 | .616 | .04 | .03 | .131 | .04 | .03 | .224 | -.02 | .03 | .406 | .02 | .03 | .480 |
| 2 | Positive PA | .04 | .02 | .107 | .00 | .02 | .858 | .03 | .02 | .206 | .03 | .02 | .214 | -.02 | .02 | .450 | .02 | .02 | .372 |
|  | *5-HTT*^b^ | -.02 | .10 | .821 | .04 | .10 | .663 | .05 | .10 | .629 | -.10 | .10 | .324 | .12 | .10 | .247 | .04 | .10 | .713 |
|  | *5-HTT*^b^ *Positive PA | **-.07** | **.03** | **.012** | .00 | .03 | .855 | ***-.06*** | ***.03*** | ***.035*** | ***-.06*** | ***.03*** | ***.020*** | .00 | .03 | .971 | ***-.06*** | ***.03*** | ***.033*** |
| 3 | Positive PA | .00 | .02 | .813 | -.01 | .02 | .717 | -.01 | .02 | .750 | -.01 | .02 | .627 | -.01 | .02 | .596 | .00 | .02 | .800 |
|  | *DRD4*^c^ | .05 | .10 | .591 | .08 | .10 | .454 | .11 | .10 | .264 | -.06 | .10 | .526 | .07 | .10 | .524 | .01 | .10 | .930 |
|  | *DRD4*^c^*Positive PA | -.03 | .03 | .267 | .02 | .03 | .496 | -.01 | .03 | .741 | -.01 | .03 | .708 | -.02 | .03 | .437 | -.03 | .03 | .205 |
|  |  |  |  |  |  |  |  |  |  |  |  |  |  |  |  |  |  |  |  |
| 1 | Deviant PA | .01 | .01 | .374 | -.02 | .01 | .113 | -.01 | .01 | .691 | .02 | .01 | .097 | .01 | .01 | .452 | **.04** | **.01** | **.007** |
|  | *DAT1*^a^ | .09 | .10 | .384 | .00 | .10 | .961 | .07 | .10 | .477 | -.03 | .10 | .749 | -.05 | .10 | .661 | -.07 | .10 | .474 |
|  | *DAT1*^a^ *Deviant PA | -.01 | .02 | .528 | -.02 | .02 | .432 | -.03 | .02 | .209 | ***-.06*** | ***.02*** | ***.018*** | .02 | .02 | .411 | ***-.05*** | ***.02*** | ***.032*** |
| 2 | Deviant PA | .01 | .02 | .387 | -.02 | .02 | .281 | .00 | .02 | .775 | .00 | .02 | .911 | ***.03*** | ***.02*** | ***.033*** | ***.03*** | ***.02*** | ***.030*** |
|  | *5-HTT* | -.02 | .10 | .844 | .03 | .10 | .764 | .04 | .10 | .662 | -.10 | .10 | .327 | .14 | .10 | .176 | .05 | .10 | .590 |
|  | *5-HTT*^b^ *Deviant PA | -.02 | .02 | .484 | -.02 | .02 | .333 | -.02 | .02 | .354 | .02 | .02 | .479 | -.03 | .02 | .114 | -.02 | .02 | .281 |
| 3 | Deviant PA | .01 | .01 | .413 | -.01 | .01 | .339 | .00 | .01 | .786 | .00 | .01 | .884 | .02 | .01 | .237 | .02 | .01 | .199 |
|  | *DRD4*^c^ | .06 | .10 | .569 | .03 | .10 | .766 | .09 | .10 | .378 | -.05 | .10 | .592 | .08 | .10 | .414 | .04 | .10 | .723 |
|  | *DRD4*^c^*Deviant PA | -.01 | .02 | .550 | -.04 | .02 | .066 | -.03 | .02 | .183 | .01 | .02 | .635 | .00 | .02 | .911 | .01 | .02 | .504 |

*Note:* ^a^ Reference group: 9-repeat absent; ^b^ reference group: short allele absent; ^c^ reference group: 7-repeat absent. PA=peer affiliation. Findings in bold are significant after correction for multiple testing (*p* < .013), findings in bold and italic are nominally significant (i.e. not significant after correction for multiple testing; *p* ≤ .05). All analyses were corrected for age, gender and collection site.

Table S4

*Mixed model analyses testing the separate main effects of maternal expressed emotions, peer affiliation and plasticity genes on neural* measures of reward sensitivity

|  |  | Reward anticipation | | | | | |  | Reward Receipt | | | | | |
| --- | --- | --- | --- | --- | --- | --- | --- | --- | --- | --- | --- | --- | --- | --- |
|  | | VS | | | vmPFC | | |  | VS | | | vmPFC | | |
|  |
|  | | *B* | *SE* | *p* | *B* | *SE* | *p* |  | *B* | *SE* | *p* | *B* | *SE* | *p* |
| Main EE effects | |  |  |  |  |  |  |  |  |  |  |  |  |  |
| 1 | Warmth | -.02 | .09 | .824 | .05 | .09 | .588 |  | -.04 | .09 | .650 | -.14 | .09 | .110 |
| 2 | Criticism | .00 | .08 | .993 | .07 | .08 | .426 |  | **.21** | **.08** | **.009** | .10 | .08 | .206 |
|  |  |  |  |  |  |  |  |  |  |  |  |  |  |  |
| Main PA effects | |  |  |  |  |  |  |  |  |  |  |  |  |  |
| 1 | Positive PA | -.01 | .01 | .676 | .01 | .01 | .679 |  | .00 | .01 | .773 | -.01 | .01 | .612 |
| 2 | Deviant PA | .00 | .01 | .970 | -.02 | .01 | .058 |  | .00 | .01 | .702 | .01 | .01 | .381 |
|  |  |  |  |  |  |  |  |  |  |  |  |  |  |  |
| Main gene effects | |  |  |  |  |  |  |  |  |  |  |  |  |  |
| 1 | *DAT1*^a^ | .01 | .11 | .930 | .07 | .11 | .544 |  | -.17 | .11 | .108 | -.01 | .11 | .958 |
| 2 | *5-HTT*^b^ | -.05 | .11 | .664 | .14 | .11 | .213 |  | -.14 | .11 | .190 | -.02 | .11 | .884 |
| 3 | *DRD4*^c^ | -.09 | .11 | .405 | -.14 | .11 | .203 |  | .10 | .11 | .349 | .06 | .11 | .559 |

*Note:* ^a^ Reference group: 9-repeat absent; ^b^ reference group: short allele absent; ^c^ reference group: 7-repeat absent. PA=peer affiliation. Findings in bold are significant after correction for multiple testing (*p* < .013), findings in bold and italic are nominally significant (i.e. not significant after correction for multiple testing; *p* ≤ .05). All analyses were corrected for age, gender and collection site.

Table S5

*Mixed model analyses testing interaction effects between plasticity genes and maternal expressed emotion or peer affiliation on neural measures of reward sensitivity*

|  |  | Reward anticipation | | | | | |  | Reward Receipt | | | | | |
| --- | --- | --- | --- | --- | --- | --- | --- | --- | --- | --- | --- | --- | --- | --- |
|  |  | VS | | | vmPFC | | |  | VS | | | vmPFC | | |
|  |
|  |  | *B* | *SE* | *p* | *B* | *SE* | *p* |  | *B* | *SE* | *p* | *B* | *SE* | *p* |
|  |  |  |  |  |  |  |  |  |  |  |  |  |  |  |
| 1 | Warmth | .04 | .11 | .735 | .05 | .11 | .644 |  | .05 | .11 | .650 | -.05 | .11 | .642 |
|  | *DAT1*^a^ | .11 | .17 | .495 | .11 | .16 | .511 |  | .12 | .17 | .484 | .16 | .16 | .330 |
|  | *DAT1*^a^ *Warmth | -.13 | .20 | .502 | -.28 | .20 | .154 |  | .03 | .20 | .890 | -.17 | .19 | .361 |
| 2 | Warmth | -.08 | .15 | .575 | .12 | .14 | .417 |  | .02 | .15 | .872 | -.06 | .14 | .659 |
|  | *5-HTT*^b^ | .04 | .16 | .791 | .14 | .16 | .379 |  | .21 | .17 | .200 | .15 | .16 | .347 |
|  | *5-HTT*^b^*Warmth | .11 | .18 | .530 | -.24 | .18 | .178 |  | .02 | .18 | .894 | -.09 | .18 | .594 |
| 3 | Warmth | .15 | .10 | .140 | .02 | .10 | .860 |  | .11 | .11 | .290 | -.09 | .10 | .381 |
|  | *DRD4*^c^ | -.08 | .16 | .610 | **.48** | **.16** | **.003** |  | -.17 | .17 | .329 | -.02 | .16 | .910 |
|  | *DRD4^c^**Warmth | **-.55** | **.19** | **.004** | -.19 | .19 | .306 |  | -.17 | .20 | .400 | -.06 | .19 | .732 |
|  |  |  |  |  |  |  |  |  |  |  |  |  |  |  |
| 1 | Criticism | -.03 | .10 | .726 | .19 | .10 | .051 |  | .04 | .10 | .723 | .07 | .09 | .440 |
|  | *DAT1*^a^ | .11 | .16 | .502 | .13 | .16 | .419 |  | .17 | .17 | .318 | .14 | .16 | .397 |
|  | *DAT1*^a^*Criticism | .14 | .19 | .478 | .12 | .19 | .530 |  | .13 | .20 | .518 | .08 | .19 | .680 |
| 2 | Criticism | -.03 | .12 | .774 | .22 | .12 | .063 |  | -.01 | .12 | .942 | .09 | .12 | .456 |
|  | *5-HTT*^b^ | .03 | .16 | .843 | .18 | .16 | .267 |  | .23 | .16 | .156 | .13 | .16 | .396 |
|  | *5-HTT^b^**Criticism | .07 | .16 | .676 | .00 | .16 | .997 |  | .15 | .16 | .355 | .03 | .16 | .862 |
| 3 | Criticism | -.04 | .10 | .665 | .16 | .09 | .076 |  | .04 | .10 | .673 | .03 | .09 | .714 |
|  | *DRD4*^c^ | -.10 | .17 | .552 | **.56** | **.16** | **<.001** |  | -.16 | .17 | .356 | .01 | .16 | .943 |
|  | *DRD4*^c^*Criticism | .14 | .18 | .453 | .29 | .17 | .087 |  | .05 | .18 | .779 | .21 | .17 | .226 |
|  |  |  |  |  |  |  |  |  |  |  |  |  |  |  |
| 1 | Positive PA | .01 | .02 | .776 | -.01 | .02 | .707 |  | .01 | .02 | .445 | -.02 | .02 | .165 |
|  | *DAT1*^a^ | .01 | .11 | .931 | -.16 | .11 | .141 |  | .08 | .11 | .489 | .02 | .11 | .874 |
|  | *DAT1*^a^ *Positive PA | .00 | .03 | .951 | .01 | .03 | .641 |  | .00 | .03 | .926 | .05 | .03 | .109 |
| 2 | Positive PA | .01 | .02 | .636 | .01 | .02 | .617 |  | .01 | .02 | .669 | -.01 | .02 | .690 |
|  | *5-HTT*^b^ | -.05 | .11 | .659 | -.14 | .11 | .201 |  | .14 | .11 | .192 | -.01 | .11 | .895 |
|  | *5-HTT*^b^ *Positive PA | -.02 | .03 | .475 | -.03 | .03 | .337 |  | .00 | .03 | .901 | .00 | .03 | .978 |
| 3 | Positive PA | -.02 | .02 | .316 | -.01 | .02 | .492 |  | .00 | .02 | .842 | -.02 | .02 | .195 |
|  | *DRD4*^c^ | -.09 | .11 | .409 | .10 | .11 | .348 |  | -.14 | .11 | .201 | .06 | .11 | .572 |
|  | *DRD4*^c^*Positive PA | .05 | .03 | .120 | .02 | .03 | .486 |  | .01 | .03 | .720 | .04 | .03 | .155 |
|  |  |  |  |  |  |  |  |  |  |  |  |  |  |  |
| 1 | Deviant PA | .00 | .01 | .764 | -.01 | .01 | .422 |  | -.02 | .01 | .128 | .02 | .01 | .197 |
|  | *DAT1*^a^ | .01 | .11 | .891 | -.16 | .11 | .134 |  | .06 | .11 | .580 | .02 | .11 | .826 |
|  | *DAT1*^a^ *Deviant PA | .02 | .02 | .523 | .01 | .02 | .546 |  | .00 | .02 | .962 | -.03 | .02 | .249 |
| 2 | Deviant PA | -.01 | .02 | .452 | .00 | .02 | .904 |  | -.03 | .02 | .099 | .02 | .02 | .355 |
|  | *5-HTT* | -.05 | .11 | .618 | -.14 | .11 | .193 |  | .12 | .11 | .264 | .00 | .11 | .975 |
|  | *5-HTT*^b^ *Deviant PA | .03 | .02 | .252 | -.01 | .02 | .720 |  | .01 | .02 | .585 | -.01 | .02 | .603 |
| 3 | Deviant PA | .00 | .02 | .966 | -.01 | .02 | .552 |  | -.02 | .02 | .147 | .02 | .01 | .164 |
|  | *DRD4*^c^ | -.09 | .11 | .404 | .10 | .11 | .349 |  | -.17 | .11 | .129 | .07 | .11 | .546 |
|  | *DRD4*^c^*Deviant PA | .00 | .02 | .908 | .02 | .02 | .504 |  | -.01 | .02 | .661 | -.03 | .02 | .201 |

*Note:* ^a^ Reference group: 9-repeat absent; ^b^ reference group: short allele absent; ^c^ reference group: 7-repeat absent. Findings in bold are significant after correction for multiple testing (*p* < .013), findings in bold and italic are nominally significant (i.e., not significant after correction for multiple testing; *p* ≤ .05). All analyses were corrected for age, gender, and collection site.

**References**

Brookes, K., Xu, X., Chen, W., Zhou, K., Neale, B., Lowe, N., et al. (2006). The analysis of 51 genes in DSM-IV combined type attention deficit hyperactivity disorder: association signals in DRD4, DAT1 and 16 other genes. *Mol Psychiatry, 11*(10), 934-953.

Greve, Douglas N., & Fischl, Bruce. (2009). Accurate and robust brain image alignment using boundary-based registration. *NeuroImage, 48*(1), 63-72.

Jenkinson, M., & Smith, S. (2001). A global optimisation method for robust affine registration of brain images. *Med Image Anal, 5*(2), 143-156.

Jenkinson, Mark, Bannister, Peter, Brady, Michael, & Smith, Stephen. (2002). Improved optimization for the robust and accurate linear registration and motion correction of brain images. *NeuroImage, 17*(2), 825-841.

Jenkinson, Mark, Beckmann, Christian F., Behrens, Timothy E. J., Woolrich, Mark W., & Smith, Stephen M. (2012). FSL. *NeuroImage, 62*(2), 782-790.

Keulers, Esther H. H., Goulas, Alexandros, Jolles, Jelle, & Stiers, Peter. (2012). Maturation of task-induced brain activation and long range functional connectivity in adolescence revealed by multivariate pattern classification. *NeuroImage, 60*(2), 1250-1265.

Landaas, E. T., Johansson, S., Jacobsen, K. K., Ribases, M., Bosch, R., Sanchez-Mora, C., et al. (2010). An international multicenter association study of the serotonin transporter gene in persistent ADHD. *Genes Brain Behav, 9*(5), 449-458.

Xu, X., Duman, E. A., Anney, R., Brookes, K., Franke, B., Zhou, K., et al. (2008). No association between two polymorphisms of the serotonin transporter gene and combined type attention deficit hyperactivity disorder. *Am J Med Genet B Neuropsychiatr Genet, 147B*(7), 1306-1309.
